# Human papillomavirus as a single infection in pregnant women from Northeastern Mexico: Cross-sectional study

**DOI:** 10.18502/ijrm.v18i2.6433

**Published:** 2020-02-27

**Authors:** Bernardo Martínez-Leal, Karla Ivette Álvarez-Banderas, Homero Sánchez-Dávila, Martha Imelda Dávila-Rodríguez, Elva Irene Cortés-Gutiérrez

**Affiliations:** ^1^Universidad de Monterrey, Vicerrectoría Ciencias de la Salud, San Pedro Garza García, México.; ^2^Department of Clinical Dysplasia, Gynecology and Obstetrics No.23 Hospital, Mexican Institute of Social Security, Monterrey, Mexico.; ^3^Universidad Autónoma de Nuevo León, Faculty of Public Health and Nutrition, Monterrey, México.; ^4^Universidad Autónoma de Nuevo León, Faculty of Biological Sciences, San Nicolás de los Garza, México.

**Keywords:** Papillomavirus infection, Pregnant women, Uterine cervical dysplasia.

## Abstract

**Background:**

The role of human papillomavirus (HPV) as single or multiple infections in pregnant women would be relevant to determine the time to progression and/or the time to regression of cervical lesions.

**Objective:**

In this preliminary study, we determined the prevalence of HPV as single or multiple infections in pregnant women from Northeastern Mexico.

**Materials and Methods:**

Samples from 31 pregnant and 62 nonpregnant women were examined between January 2015 and November 2015 at UMAE-23 of the Instituto Mexicano del Seguro Social (IMSS). The samples of cervicovaginal exudate were obtained for HPV DNA detection using the INNO-LiPA test, and HPV infections were analyzed as single or multiple infections. Participants completed a questionnaire on sociodemographic, gynecological, obstetric, and sexual behavior characteristics.

**Results:**

The mean age of the pregnant women was 25.7 ± 4.8 yr, with an average time of pregnancy of 6 ± 1 months at the time of the study. With respect to age, parity, smoking history, or oral contraceptive use no statistically significant differences between the two studied groups was observed. The HPV infection was 2.7 times higher in pregnant women (35%) than in the control group (13%). In total, 78% of the pregnant women who were HPV-positive presented with single infections compared with 28% of the nonpregnant women.

**Conclusion:**

A higher prevalence of HPV as a single infection was found in this sample of pregnant Mexican women. Follow-up is necessary to evaluate the persistence or regression of the infection.

## 1. Introduction

In Mexico, cervical carcinoma is the second most common neoplasia and the second most frequent cause of death by cancer in women (1). Human papillomavirus (HPV) types 16, 18, 31, 45, and 58 are the most frequent in cervical samples from Mexican patients (2). Many factors can increase the risk of HPV infection; for example, sexual behavior, number of sexual partners, vaginal infection, and early onset of sexual activity. Diet, smoking, genetic factors, and immune suppression are also related to HPV infection (3-6). Studies have reported that pregnancy affects the immune system of the host and alters seroreactivity to HPV infection (7, 8). Another important factor that should be considered in HPV-infected patients is the genotype distribution between single and multiple infections. Recently, Aguilar- Lemarroy and co-workers reported a higher prevalence of the HPV genotype as a single infection in Mexican patients with cervical cancer (2). Few studies have addressed the role of HPV as single or multiple infections in pregnant women. However, this information would be relevant to determining the time to progression and/or the time to regression of cervical lesions.

Thus, this case-control study was conducted to investigate the presence of HPV as single or multiple infections in cervical samples from pregnant and nonpregnant women using the INNO-LiPA test.

## 2. Materials and Methods

### Subjects and sample collection

Thirty-one pregnant women between age 19 and 27 years in the third trimester of pregnancy and 62 non-pregnant women (as controls) within a similar age range (18-28 yr) were prospectively enrolled in this cross-sectional study from January 2015 to November 2015.

All pregnant women who visited the Gynecology and Obstetrics Department of UMAE-23, Instituto Mexicano del Seguro Social (IMSS), Mexico, for the first time during pregnancy were invited to join the study. A gynecologist explained the aim of the study, the procedures, and the complications involved. Control samples were obtained from women attending early cancer detection programs at the same hospital. This was followed by an interview conducted by research assistants, in which all the participants responded to a questionnaire respecting reproductive history and sexual behavior.

None of the women included in the study presented cervical lesions, as assessed by clinical examination of the cervix, cytology, and colposcopy. Women with immunosuppressive diseases, as hepatitis C, hepatitis B, HIV, and diabetes mellitus, were eliminated from the study.

Cervical samples were obtained with a cytobrush in the course of gynecological exploration. The cytobrush was inserted into the endocervical canal, rotated 3 to 5 times, placed into the transport medium (Preserv-Cyt solution; Hologic, Bedford, MA), and immediately sent to the Laboratory of Molecular Cytogenetics of Centro de Investigación Biomédica del Noreste, IMSS, Mexico. Cervical scraping samples were frozen at -20°C until DNA extraction. DNA was extracted using a Wizard Genomic DNA Purification Kit (Promega, Madison, WI). The DNA quantity and quality were verified using a NanoDropⓇ Jenway kit (Genova Life Science, OSA, UK).

### Detection/genotyping

The INNO-LiPA HPV genotyping test (Innogenetics N.V., Ghent, Belgium) directs the amplification of a 65 bp region of the HPV L1 gene and allows the identification of 28 anogenital HPV genotypes: twelve high-risk (HR) HPVs, one probable HR HPV, seven possible HR HPVs, and eight low-risk HPVs (9). Two internal controls and two positive controls (both 65 bp products from the HPV L1 region) were included. Results from specimens where the controls failed to amplify were excluded from the study. The INNO-LiPA test permits the simultaneous detection of multiple genotypes in a single sample.

### Ethical consideration

Written informed consent was obtained from all participants and the study protocol was reviewed and approved by the Ethics and Scientific Committee of IMSS (Code: 293539).

### Statistical analysis

The risk of HPV infection associated with pregnancy [odds ratio (OR) and 95% confidence interval (CI)] was estimated by logistic regression, P < 0.05 using the IBM SPSS Statistics, version 22 (SPSS, Inc., Chicago, IL).

## 3. Results

No statistically significant differences were found between the two groups regarding age, (age mean, 25.7 ± 4.8 years in for pregnant women vs 28.6 ± 5.1 years in nonpregnant), parity, smoking during pregnancy, oral contraceptive use, and history of sexually transmitted infections (Table I).

Among the 31 pregnant women studied here, 11 (35%) were HPV-positive. Of these, eight (73%) were infected with a single genotype and three (27%) were infected with multiple genotypes. HPV-16 was detected in six pregnant women (54%; five as a single infection and one as multiple infections). Among the 62 nonpregnant women, eight were HPV-positive (12%). Of these, three (36%) were infected with a single genotype and five (64%) were infected with multiple genotypes. The HPV-44 and HPV-52 genotypes were the most prevalent in all cases involving multiple infections (Table II).

A multivariate analysis showed that pregnancy was associated with an increased risk for multiple HPV infection (OR, 2.75; 95% CI, 1.15-6.59) and for single HPV infection (OR, 4.6667; 95% CI, 1.0814-20.1388).

**Table 1:** Clinical characteristics of the pregnant and non-pregnant women


	**Non-pregnancy**	**Pregnancy**
**Number of studied women**	62	31
**Age (yr) (X ± SD)**	28.6 ± 5.1	25.7 ± 4.8
**Parity (X ± SD)**	1.9 ± 2.4	2.3 ± 2.1
Current smokers *(%)	23	29
**Oral contraceptive ${}^{+}$(%)**	18	24
**Sexually transmitted infections (%)**	11	9
X ± SD = Mean ± standard deviation, *Current smokers were defined as women who had smoked at least 100 cigarettes in their lifetime and were smoking at the time of the visit, **${}^{+}$**Women who had used oral contraceptives for more than one year		

**Table 2:** HPV genotype distribution (single and multiple) in pregnant and non-pregnant women


**HPV-positive **		**Pregnancy (%)**	**Non-pregnancy (%)**
**Single**			
	**11**	1	**-**
	**16**	5	**-**
	**18**	1	**-**
	**6**	1	1
	**52**	- 1
	**39**	- 1
	**Sub-total**	8 (72.7)	3 (37.5)
**Multiple**			
	**6, 11, 39**	1	**-**
	**6, 11, 58**	1	**-**
	**11, 16, 31, 33, 44, 51, 52**	1	**-**
	**16, 26, 31, 33, 44, 51, 52, 54, 69-71, 82**	- 1
	**44, 52, 58**	- 1
	**51, 82**	- 1
	**44, 52, 58, 69-71**	- 1
	**16, 31, 33, 44, 52, 54**	- 1
	**Sub-total**	3 (27.3)	5 (62.5)
**TOTAL**		11 (100)	8 (100)
HPV-low risk: 6, 11, 44, 54; HPV-high risk: 16, 18, 31, 33, 35, 39, 51, 52, 58, and 8.2; HPV-possible high risk: 26, 69-71 For HPV infection (OR, 2.75; 95% CI, 1.15-6.59), For single HPV infection (OR, 4.6667; 95% CI, 1.0814-20.1388)			

## 4. Discussion

In this preliminary study, we determined the prevalence of HPV as single or multiple infections in pregnant women from Northeastern Mexico. Our findings revealed a significant increase in HPV prevalence among pregnant women (35%) compared with nonpregnant women (13%) (OR = 2.7).

Studies comparing the distribution of HPV types in pregnant and nonpregnant women are scarce in the Mexican population. Hernández-Girón *et al.* (10, 11) reported a higher prevalence of HR HPV infection in pregnant women compared with nonpregnant women, with detection rates of 37.2% (95% CI, 31-43) and 14.2% (95% CI, 12-16), respectively. Previous studies have reported similar findings (12-14).

Liu *et al*. (15) performed a meta-analysis of the risk of HPV infection in pregnant women from Asia, Europe, and North America and found a significantly increased risk among pregnant women (16%, 13%, and 30%, respectively), with a general OR of 1.42.

In contrast, Nobbenhuis (16) found no difference in HPV prevalence between pregnant and nonpregnant women. Such discrepancies may have arisen from the HPV detection method used and the number of detectable HPV genotypes. In our study, we used the INNO-LiPA test, which is able to identify 28 anogenital HPV genotypes and includes two internal controls and two positive controls. The INNO-LiPA test permits the simultaneous detection of multiple genotypes in a single sample.

Previous study reported a dependence of HPV prevalence on age (11), parity, smoking history, oral contraceptive use, and history of sexually transmitted infections. In our study, the groups under study were homogeneous in terms of these factors (17-19).

A possible explanation for the higher prevalence of HPV found in pregnant women respect to nonpregnant women is based that immunosuppression occurs during pregnancy because of the reduction of cellular immunity (7). It has been suggested that the high levels of estrogen and progesterone that occur during pregnancy decrease the cellular immunity, which is crucial for resolve the HPV infection. Our previous results showed that a high prevalence of HPV-52 and HPV-44 was involved in multiple infections in nonpregnant women (2). In contrast, a high prevalence of HPV-16 as a single infection was observed in pregnant women.

Interestingly, our results revealed that 78% of the pregnant women who were HPV-positive presented with a single infection compared with 28% of the nonpregnant women.

The transcriptional promoter of the E6/E7 oncogenic region of HPV-16 contains a steroid-hormone-receptor-binding element that activate HPV E6/E7 transcription, suggesting a hormonal stimulation effect on HPV replication. These observations indicate that pregnancy may have an effect on the patient's capacity to eradicate multiple HPV infections (20). Yamasaki (21) reported that accelerated clearance of HPV may be caused by the normalization of the host's immune system during the third trimester of pregnancy. Thus, the presence of HR HPV in the late period of pregnancy can be considered as being persistent and, therefore, represents a risk factor for neoplastic lesions.

In conclusion, a higher prevalence of HPV as a single infection in pregnant women was found, despite the number of women included in the study was relatively small. Further investigations are necessary to establish the biological role of HPV as a single infection and its participation in the development of precancerous lesions or cervical carcinoma.

##  Conflict of Interest

The authors declare that there is no conflict of interest regarding this study.
